# Prevalence of Pulmonary Tuberculosis in Diabetic Patients: Epidemiology, Immunological Basis, and Its Amalgamated Management

**DOI:** 10.7759/cureus.31321

**Published:** 2022-11-10

**Authors:** Akriti Sinha, Abhishek Joshi

**Affiliations:** 1 Community Medicine, Jawaharlal Nehru Medical college, Datta Meghe Institute of Medical Sciences, Wardha, IND; 2 Community Medicine, Jawaharlal Nehru Medical College, Datta Meghe Institute of Medical Sciences, Wardha, IND

**Keywords:** dm-tb patients, immunity impairment, glycemic control, diabetes, tuberculosis

## Abstract

Tuberculosis (TB) is one of the most widespread and infectious diseases in the world, which is brought on by *Mycobacterium tuberculosis* (MTB). Most infection lacks traditional signs. Latent TB is the name given to this ailment. Of these latent infections, 10% become active and cause illness. Fever, night sweats, a prolonged cough with blood-containing mucus, and weight loss are common signs of active TB infection. Diabetes, on the other hand, is a group of metabolic illnesses characterized by elevated serum glucose levels. It is a chronic metabolic condition brought on by a deficiency in insulin secretion or resistance. It is of two types, that is, type 1 and type 2. Among all the cases of diabetes, the occurrence of type 2 is more common and less fatal than type 1. The prevalence of diabetes is currently increasing in low- and middle-income nations. As both diabetes and TB come under the most widespread chronic condition; therefore, their combined effect is evaluated. In recent years, the higher occurrence of TB in patients with hyperglycemia has come to light. People with elevated blood glucose levels exhibit several risk factors that make them more vulnerable to contracting TB. This review provides information on epidemiological data about the prevalence of TB in patients with hyperglycemia. In addition, this paper discusses the immunological underpinnings of TB development in patients with diabetes mellitus and how glycemic management reduces the risk of TB infection. It illustrates how the clinical signs and radiographic evidence of TB differ between people with diabetes and healthy people and mentions diabetes and TB combined management.

## Introduction and background

*Mycobacterium tuberculosis* (MTB) is the microorganism that causes the highly contagious disease tuberculosis (TB), which mostly affects the lungs. It may also impact the colon, meninges, bones, joints, lymph nodes, skin, and other body tissues. Two types of TB are known. One is bovine TB, in which the cattle are affected, which can sometimes be transmitted to humans. Another is human TB affecting human beings. In human TB, pulmonary TB is a major and serious health issue affecting the population worldwide despite the availability of efficient and compelling drugs and vaccines. Geographically, the highest number of cases taken down was from Southeast Asia (44%), Africa (25%), and Western Pacific (18%). India accounts for 26% of cases and is the utmost burden country, followed by Indonesia (8.5%) and China (8.4%). In recent years, the occurrence of TB has seen a downfall in developed countries, probably due to proper management. Still, the prevalence is high in countries having an increased number of HIV cases, a high number of malnourished children, poor living conditions, and poor TB control and management [1]. On the other hand, diabetes, a cluster of metabolic disorders depicted by increased serum glucose levels, is an iceberg disease resulting from a defect in insulin resistance and/or secretion. The preponderance of diabetes is now escalating in weak economies. There are two types of diabetes known, that is, type 1 and type 2. Type 2 diabetes (non-insulin-dependent diabetes mellitus [DM]) has a higher prevalence than type 1 diabetes, as about 95% of the cases belong to the type 2 category [2]. Type 1 DM is an immune-mediated disease occurring due to the destruction of beta cells present in the pancreas, which is responsible for the production of insulin [3]. Type 2 DM (T2DM) occurs because insulin release is desensitized and insulin resistance is elevated as a result of genetic and environmental factors, with obesity being one of the major factors [4]. The combined effect of DM and TB has been known for many years [1].

## Review

Epidemiology

T2DM, a hyperglycemic disorder, has started affecting low- and middle-income countries due to rapid development and changes in food habits, with its strong prevalence in countries with high-socioeconomic status [5]. According to records, about 80% of the population with DM resides in low- and middle-income countries [6]. On the other hand, TB is an infection generally seen in overcrowded, poorly nourished countries, which can be well equated with middle- and low-income countries. The combined occurrence of these two diseases was seen in developing countries, which enthralled many epidemiologists toward the correlation between these two diseases. The increased incidence rate of DM in high-risk TB countries such as Sub-Saharan Africa has also pointed toward the fact that this hyperglycemic disorder may be the risk factor for an increased prevalence of infection caused by MTB [7]. South India, The Pacific Islands, and Mexico are the regions with an increased incidence of TB in people having T2DM [8]. The World Health Organization (WHO) has also suspected that control of TB is being compromised by the increasing number of diabetics in the world, which emphasizes the double burden of TB and diabetes [8]. In a recent study covered in Chennai, India, among the newly diagnosed TB cases, 54% had T2DM and only 25% had normal blood glucose levels [9]. DM was reported in 40% of the newly diagnosed cases of TB in the Pacific Islands [10]. Even the Texas-Mexico border region said that 25% of the newly diagnosed cases of TB were hyperglycemic [11]. All these studies, therefore, demand proper screening of TB in diabetic patients to reduce the prevalence. However, the degree of increased risk of TB and the range of treatment failure in people with diabetes is unclear [6].

DM is a risk factor for a rise in the reactivation of TB

DM, a metabolic disorder, is a significant risk factor for relapsing latent TB infection among all the identified risk factors [3]. Many unconventional clinical presentations and radiographic findings can vary in nondiabetics and diabetics affected by pulmonary TB. Diabetes can precipitate the risk of developing TB through various mechanisms. Studies have revealed that hyperglycemia makes patients vulnerable to infections by decreasing cell-mediated immunity. There is a case-control study showing the weakening of immunity because of hyperglycemia, which is responsible for converting latent TB to an active infection [12]. It has been estimated that there is a 3.1% more chance of getting infected with TB in patients having increased blood sugar than in individuals with normal blood sugar levels. A cohort study in Chile on diabetics showed that insulin-dependent diabetics are at more risk for contracting TB than non-insulin-dependent diabetics [1]. As stated in the survey by Lee et al. [12], the younger population with DM are more receptive to reinfection than relapse. In contrast, the elderly population is more susceptible to relapse of TB than reinfection [12]. As per TB treatment, the time taken for negative sputum culture is more in people with diabetes than in nondiabetics. It is also seen that the drugs used to treat TB worsen the blood glucose level. Sulfonylureas, an oral hypoglycemic medication, also shows an alteration in its metabolism because of the use of rifampicin [1]. Hence, early diagnosis of TB in diabetics is very important to minimize the complications.

Immunological basis of development of TB in diabetics

A study conducted by Ayelign et al. [3] showed that both innate immunity and acquired immunity are impaired in people with diabetes having TB infection.

Impairment of Innate Immunity

The metabolic changes brought on by DM cause neutrophils, macrophages, natural killer cells, and other innate immunity-related cells to function less effectively [13]. The alveolar macrophages engulf the mycobacterium entering the lungs, and this induces activation of innate immunity of the body, which, in response, causes the accumulation of myeloid cells in the alveoli [14]. In a study done on diabetic mice, increased expression of CCR2 was found to hamper the entry of monocytes into the lungs and decrease the expression of CD14, thus reducing the phagocytic action of the immune cells and increasing the vulnerability of individuals to TB infection [15]. It is also known that macrophages are activated by interferon-gamma whose release is regulated by IL-1beta, IL-12, and IL-18 secreted from antigen-presenting cells. T2DM deteriorates the activation of alveolar macrophages with interferon-gamma by lowering the release of IL-1beta, IL-12, and IL-18 from antigen-presenting cells [16]. Among all the immune cells of innate immunity, neutrophils reach the site of infection first and cause the accumulation of other cells by secreting cell signaling proteins, namely, cytokines and chemokines [17].

Impairment of Acquired Immunity

T helper cells 1 (Th1) are crucial to the host's defensive system because of acquired immunity. The growth and multiplication of Th1 need cytokine IL-2, and Th1 is responsible for the generation of interferon-gamma, which worsens the nitric oxide (NO)-dependent death of macrophages [18]. In individuals affected by DM, the activity of Th1 is hampered, which is responsible for the increased susceptibility of these individuals to MTB infection [19]. Studies have shown that increased production of Th2 cells secreting IL-4 in TB patients with diabetes has a deteriorating effect on the production of Th1 and Th17 cells. A review of the immunological impact of diabetes on TB conveys that the ratio of Th1 to Th2 was found to be lesser in diabetic TB patients than in nondiabetic TB patients [3]. The release of anti-inflammatory cytokines, namely, transforming growth factor-beta (TGF-beta) and IL-10, from regulatory T-cells were increased in the diabetic population compared with the nondiabetic population, which thus was responsible for the interference in the Th1 and Th2 cytokine production [20]. The part of antibodies in the patients susceptible to TB is not clear. According to many studies, the absence of antibodies against lipoarabinomannan present in the mycobacterial cell wall has a strong association with increased vulnerability to TB infection [21]. Moreover, many mechanisms, like the binding of opsonins with the foreign pathogen, the rise of calcium-mediated macrophage signaling, and the activation of the complement system provide antibody-mediated protection against TB infection [22].

**Figure 1:**
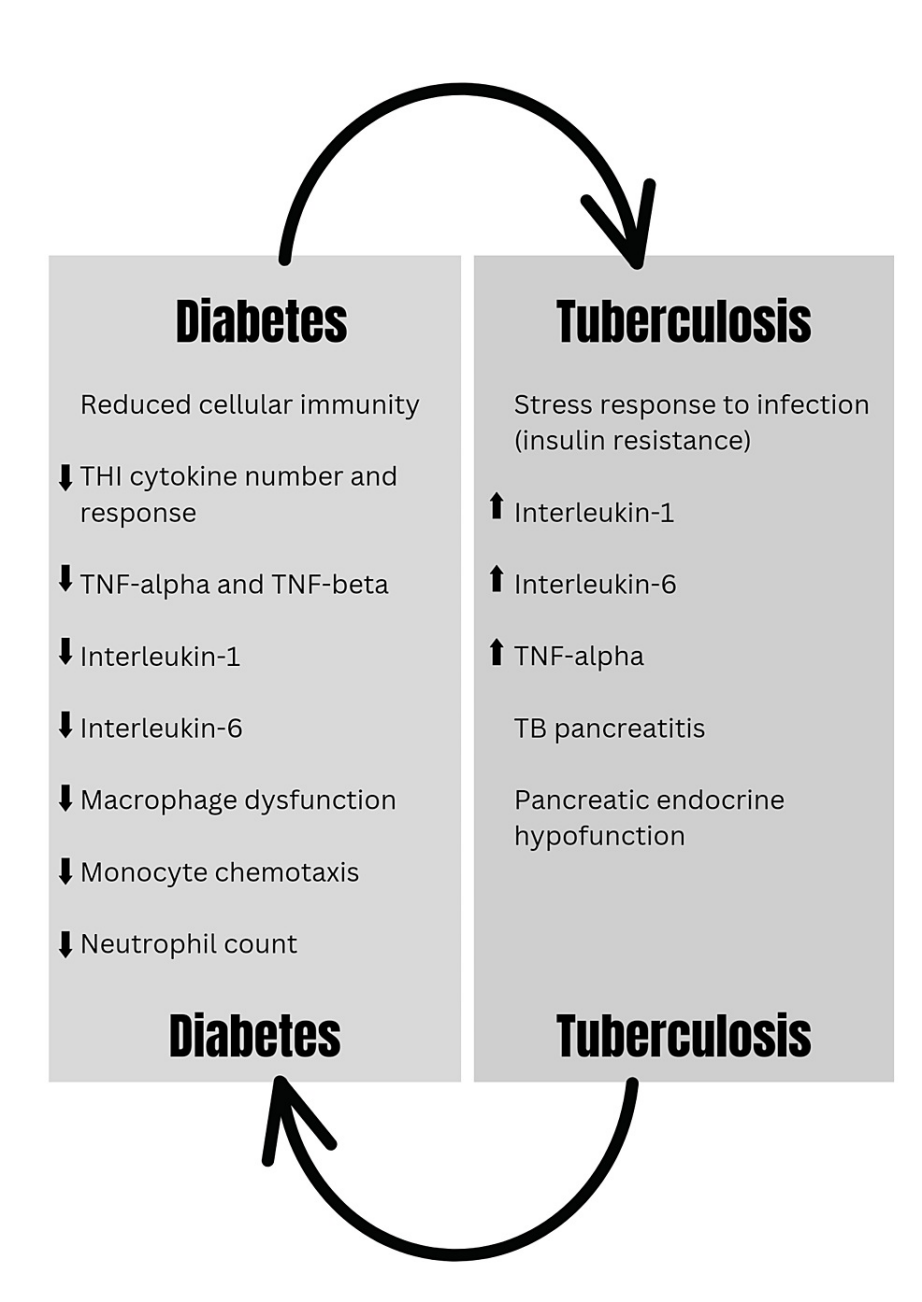
Correlation between diabetes and TB. TB, tuberculosis; TNF, tumor necrosis factor; Th1, T helper cells 1 Source: [17].

A diagrammatic representation of the correlation between diabetes and TB is given in Figure 1 [17].

Is prediabetes also a risk factor of rise for relapse of TB?

Prediabetes, also called impaired glucose tolerance, is a condition reflecting high blood sugar levels, but not high enough to be considered in the type 2 diabetes category. A fasting plasma glucose level below 99 mg/dL is normal, between 100 and 125 mg/dL indicates a prediabetic condition, and above 126 mg/dL indicates diabetes. A correlation between prediabetes and increased risk of TB was tried to be established, although due to limited evidence, this fact is not proved yet. A cross-sectional survey and research on refugees in the United States having latent TB showed that 39.1% of the refugees were prediabetics, which raised a question among epidemiologists about the association of prediabetes with an increased risk of TB infection [6]. An Indonesian study also described a notable interrelation between impaired fasting plasma glucose and the occurrence of TB [6]. The association of prediabetes with a high risk for diabetes is also mentioned in some studies, but the evidence provided is not sufficient to prove the fact.

Obesity and DM-TB

Obesity, that is, a BMI of more than 30 kg/m^2^, is a significant risk factor for TB infection because obesity simultaneously increases the risk for diabetes, but overweight, that is, BMI between 25 and 30 kg/m^2^ without diabetes, reduces the risk of TB infection [23]. Reduced vitamin D level was also thought to be the risk factor because supplementation of vitamin D in TB-DM patients improved the treatment outcome, and sufficient levels of vitamin D were found in TB-DM patients who showed a positive response to the treatment [24].

How glycemic control affects TB

For a long, people with DM and TB have been linked [1]. Numerous studies have demonstrated that DM affects treatment outcomes and might lead to infection relapses [1]. It is an essential risk factor for the development of TB. Patients with uncontrolled type 2 diabetes are more likely to acquire pulmonary TB than those with well-controlled blood sugar levels [25]. Based on a chest radiograph, a diabetes survey was conducted in Philadelphia by Dooley and Chaisson who found that the prevalence of pulmonary tuberculosis was two times higher in diabetics than in nondiabetics [1]. Epidemiologists typically concentrated on active TB while researching the prevalence of TB in people with diabetes. In research conducted in a general care clinic in Spain, of 163 diabetics, 69 (42%) responded positively to a tuberculin skin test, raising the possibility that latent TB is more common in diabetic patients. Numerous other longitudinal cohort studies have produced comparable findings [26]. A five-year survey of 42,000 seniors in Hong Kong showed that diabetic patients with hemoglobin A1c (HbA1c) levels higher than 7% were at a higher risk of developing active TB than nondiabetic patients [27]. Although UK-based cohort research indicated that those with uncontrolled DM did not have a greater danger of developing TB than those whose level of blood sugar is under control [28], another analytical investigation from Denmark showed the same: HbA1c levels do not indicate how widespread TB is. Therefore, it remained unknown whether the blood level of HbA1c affects TB in patients with diabetes. However, a meta-analysis conducted by Chen et al., which included data from 17 research, revealed a link between high TB prevalence and elevated HbA1c levels in the blood [29]. This suggested mandatory TB screening for people with inadequate glucose regulation.

How to reduce the incidence of DM-TB: Is DM prevention worth it?

Failure of DM prevention has focused the concept on its ways of prevention and treatment to reduce the incidence of TB. A program named Diabetes Prevention Program was a lifestyle change program initiated to prevent diabetes by changing the lifestyle of people and by using metformin. This program showed a fall in several cases of diabetes but for only a short period. After a long-term follow-up, it was disclosed that this program only delayed the onset of diabetes but could not prevent it [30]. This program also required a lot of financial support, which is impossible in low-income countries. Therefore, early identification and management of blood glucose levels, together with lifestyle changes, can reduce the chance of getting TB in diabetics [31].

Radiographic findings in DM-TB patients

The radiographic finding varies with the duration of illness and host immunity. In a study by Dooley and Chaisson, lower lung involvement was seen in people with diabetes with TB, while the nondiabetics showed infiltration in the upper lobe [1]. Another study conducted in the 1970s and 1980s also showed similar findings, which made people believe that pulmonary TB in people with hyperglycemia shows a typical radiographic pattern with involvement of the lower lung [1]. This point was important from a clinical point of view because pneumonia or lung cancer also offers a similar kind of lower lung involvement.

Amalgamated management of diabetes and TB

India is the highest-burden country with coexisting cases of diabetes and TB worldwide [32]. Hence, a patient diagnosed with TB should be screened for DM, as recommended by WHO [33]. But there are no international guidelines or national programs for the amalgamated management of diabetic patients affected by TB [34]. It is reported that if a patient affected by TB is diabetic, then it will hamper their treatment outcome as well as can cause a relapse of the disease. However, certain observational studies show improvement in treatment outcomes in DM-TB patients if the duration of treatment is lengthened [35]. Reduced concentrations of rifampicin, isoniazid, and other antitubercular drugs in DM-TB patients are also found in some studies [36], which put weight on increasing the dosage of medicines to get the desired results. Some evidence demonstrates diabetes as a risk factor for drug-resistant (DR) TB, but there are no such guidelines for using second-line medications in these patients. Also, there is delayed sputum conversion and severe clinical symptoms like high frequency of cavities as seen in chest X-rays in these patients. In a study, diabetes was induced in the study animals, which showed increased shedding of MTB in the airway, giving an idea of increased bacterial load [37]. Also, the hyperglycemic guinea pigs showed a rise in tumor necrosis factor (TNF) alpha and IL-1 beta along with elevated bacterial load, which was thought to be the reason for the failure of antitubercular treatment (ATT) in TB patients with diabetes [38]. It was also postulated that this increased bacterial load was responsible for altering the absorption, distribution, metabolism, and excretion of antitubercular drugs [39]. In a study by Lee et al., the treatment of DM-TB patients with metformin showed an increase in sputum culture conversion rate as compared to patients who were not treated with metformin [40]. Among various treatment modalities, host-directed therapeutic (HDT) strategies are looked for as an adjunctive treatment to harmonize the immunity of the individual, which will result in early pathogen removal and a shortening of treatment duration [41]. Yet another experimental study done by Singhal et al. showed suppression of MTB with the use of metformin [42].

However, more studies are required to determine the exact role of metformin in cavitary TB patients as, until now, it cannot be used as general adjuvant therapy for diabetic patients with coexisting TB infection. Apart from the glycemic levels, other things are also present in DM-TB patients, which should be considered, and proper treatment and management should be provided. For example, an increased mortality rate was found in DM-TB patients from cardiovascular disease, as mentioned in studies held in Taiwan and Tanzania [43].

## Conclusions

This review focuses on the increasing prevalence of pulmonary TB in diabetics, thus highlighting the importance of early screening and amalgamated management of diabetes and TB. It also highlights the immunological impairment seen in diabetics, which makes the individual vulnerable to TB infection. More epidemiological studies should be carried out to establish a more strong correlation between these two diseases and to determine the exact pathophysiology responsible for it. However, no adequate guidelines for the amalgamated management of DM and TB are there, but controlling blood glucose levels reduces the risk. It is one of the major steps taken to reduce the incidence of pulmonary TB in diabetics. Other associated factors in DM-TB patients should also be considered for proper management, giving equal weightage to all. Overall, still many studies are required and should be carried out to verify the use of escalated and prolonged ATT in TB patients with associated diabetes. All possible investigations and interventions should be used to check and reduce DM in DM-TB patients, like glycemic control, management of cardiovascular risk, and periodic screening for DM.
